# Despite inducing antioxidant regulation, superoxide dismutase deficiency makes *Escherichia coli* more sensitive to hydrogen peroxide

**DOI:** 10.3389/fmicb.2026.1793871

**Published:** 2026-03-10

**Authors:** Yuejuan Nong, Jiaxin Qiao, Yixuan Zhao, Jingjing Wang, Li Xin, Weijie Wang, Weiwei Zhu

**Affiliations:** 1 State Key Laboratory of Vaccines for Infectious Diseases, Xiang An Biomedicine Laboratory, National Innovation Platform for Industry-Education Integration in Vaccine Research, Department of Laboratory Medicine, School of Public Health, Xiamen University, Xiamen, China; 2 Inner Mongolia Key Laboratory for Molecular Regulation of the Cell, School of Life Sciences, Inner Mongolia University, Hohhot, China; 3 Research Center for Clinical Medicine, The First Affiliated Hospital of Kunming Medical University, Kunming, Yunnan, China; 4 Department of Clinical Pharmacy, The Affiliated Hospital of Yunnan University, Kunming, China

**Keywords:** *Escherichia coli*, hydrogen peroxide, metabolic reprogramming, oxidative stress regulation, superoxide dismutase

## Abstract

Superoxide is a toxic byproduct of aerobic cellular respiration. The cellular regulation of bacterial responses to superoxide stress remains incompletely understood. The present work established an *Escherichia coli* cell model for superoxide stress by deleting superoxide dismutase (SOD) SodA and SodB. Proteomic analysis revealed that SOD deficiency not only induced high expression of the oxidative stress regulator SoxSR but also upregulated the catalase KatE and the organic peroxidases Tpx and BtuE, suggesting that SOD deficiency leads to the subsequent production of multiple reactive oxygen species. Further analysis of central carbon metabolism networks showed that SOD deficiency suppressed oxidative phosphorylation, thereby reducing superoxide production. SOD defects stimulated the pentose phosphate pathway (PPP) and its downstream pathways involved in histidine and phenylalanine synthesis, as well as fatty acid degradation pathway. SOD deficiency rendered *E. coli* more sensitive to the lethal effects of exogenous hydrogen peroxide. CRISPR-mediated deletion of *zwf* to block PPP, deletion of *hisD* and *pheA* to disrupt histidine and phenylalanine synthesis, or deletion of *fadE* to block fatty acid degradation, all increased the SOD mutant’s sensitivity to hydrogen peroxide. The absence of *fadE*, rather than *hisD* or *pheA*, further reduced the survival of *zwf*-SOD mutant under H_2_O_2_ killing. These data indicate that the PPP and fatty acid degradation pathways help SOD-deficient cells respond to oxidative stress. Overall, our findings offer new perspectives on bacterial defenses against oxidative stress and survival strategies.

## 1 Introduction

Oxygen is essential for the survival of aerobic organisms; however, every coin has two sides. While aerobic respiration effectively produces energy, it also generates toxic metabolic byproducts, such as superoxide (O_2_^–^) (Imlay, 2008). In response to oxidative stress, organisms have evolved superoxide dismutase (SOD) (McCord and Fridovich, 1969; McCord et al., 1971; Case, 2017), which rapidly catalyzes the disproportionation of superoxide radicals into hydrogen peroxide and oxygen (Culotta, 2000; Imlay, 2008). SodA (manganese-dependent) and SodB (iron-dependent) are located in the cytoplasm and are the two primary SODs responsible for scavenging intracellular superoxide in *Escherichia coli* (Britton and Fridovich, 1977; Hopkin et al., 1992). SodC (copper-/zinc-dependent) is localized in the periplasm and is induced during the stationary phase (Fridovich, 1995; Gort et al., 1999). Therefore, the *sodA*-*sodB* double-deletion mutant is commonly used as a cellular model to investigate how *E. coli* responds to superoxide stress during growth. A slight decrease in SOD levels in *Escherichia coli* causes cellular damage (Imlay and Fridovich, 1991; Gort and Imlay, 1998). Previous studies have showed that targeting metabolic enzymes containing iron-sulfur clusters is one way superoxide mediates cellular toxicity (Brown et al., 1995; Benov and Fridovich, 1999). The attack on iron-sulfur clusters by superoxide releases iron ions (Gardner and Fridovich, 1991a,b; Flint et al., 1993), leading to iron-mediated cellular damage (Liochev and Fridovich, 1994; Keyer et al., 1995; Keyer and Imlay, 1996). This highlights the role of iron reactions in superoxide-mediated lethality. Additionally, osmotic pressure stabilizers can alleviate growth defects caused by SOD deficiencies (Imlay and Fridovich, 1992; Benov et al., 1996), suggesting that envelope damage is also a component of superoxide lethality. However, from a chemical perspective, superoxide does not react significantly with amino acids, nucleic acids, lipids, or carbohydrates (Bielski and Richter, 1977; Fitzsimons, 1979; Sawyer and Valentine, 1981; Fee, 1982; Gu and Imlay, 2013). Exploring the mechanisms of superoxide-induced cell damage has been an exciting endeavor, and the revelations to date may not capture the full picture. Omics technologies offer advantages for the systematic analysis of cellular and molecular response mechanisms. Proteins are the performers of biological functions. This work used proteomics to analyze the response of *E. coli* following *sodA*-*sodB* deletion, aiming to enhance understanding of bacterial oxidative stress defense and survival strategies.

## 2 Results

### 2.1 The induction of anti-oxidative strategies indicates a rise of oxidative stress in the SOD mutant

A double deletion of *sodA*-*sodB* (SOD deficiency, SOD mutant) in *E. coli* considerably slows the growth rate (the doubling time increases by 50.8 min compared to wild-type cells) and inhibits DNA replication initiation (Qiao et al., 2025a). This cellular response is linked to increased superoxide and oxidative stress due to SOD deficiency (Imlay and Fridovich, 1991), as the antioxidant N-acetylcysteine (NAC) reverses the delay in replication initiation caused by SOD deficiency (Qiao et al., 2025a). In this work, based on proteomics analysis, we found that SOD defects led to a significant upregulation of SoxRS expression rather than OxyR (Supplementary Figure 1A). This is consistent with previous reports of SoxRS association with superoxide levels (Greenberg et al., 1990; Tsaneva and Weiss, 1990; Gu and Imlay, 2011). The deficiency of SOD theoretically reduces hydrogen peroxide from superoxide hydrolysis (Culotta, 2000). Interestingly, the expression of the stress-induced catalase KatE (Schellhorn and Hassan, 1988; Tanaka et al., 1997) was increased in the SOD-deficient mutant compared with that of the wild type cells, and there was no significant difference in the expression of the constitutive catalase KatG and the alkyl hydroperoxide reductase AhpCF (Supplementary Figure 1B). Previous studies have shown that catalase is the primary scavenger at high hydrogen peroxide concentrations (Seaver and Imlay, 2001a,b). This suggests that SOD deficiency may increase intracellular hydrogen peroxide levels in *E. coli*. Additionally, the levels of lipid hydroperoxide peroxidase Tpx and glutathione peroxidase BtuE were elevated during SOD deficiency (Supplementary Figure 1B). BtuE is a non-specific peroxidase that combines thioredoxin and glutathione to decompose organic hydroperoxides (Arenas et al., 2010, 2011). Glutathione is a well-known endogenous antioxidant (Forman et al., 2009; Averill-Bates, 2023). While the expression of the synthetase GshAB did not change significantly after the deletion of *sodA*-*sodB*, the glutathione-glutaredoxin redox reactions protein GrxAB (Ritz and Beckwith, 2001; Ortenberg and Beckwith, 2003), which is involved in redox regulation, increased (Supplementary Figure 1C). Overall, our findings solidify the observation that SOD deficiency induces oxidative stress (Imlay and Fridovich, 1991; Gort and Imlay, 1998).

### 2.2 SOD deficiency inhibited oxidative phosphorylation

**FIGURE 1:**
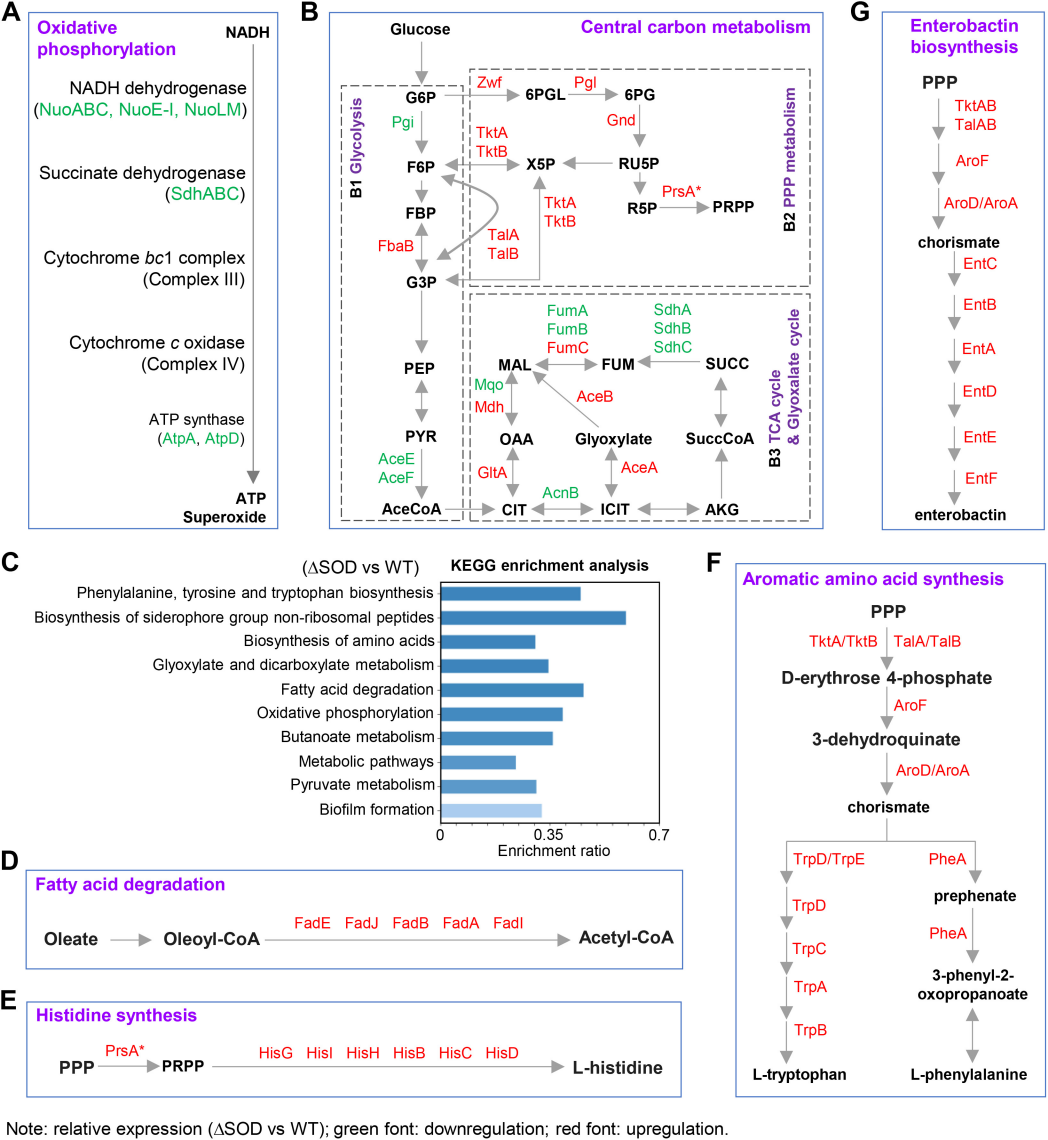
The effects of SodA-SodB deficiency on oxidative phosphorylation **(A)**, central carbon metabolism **(B)**, fatty acid degradation **(D)**, histidine synthesis **(E)**, aromatic amino acid synthesis **(F)**, and enterobactin synthesis **(G)** in exponentially growing *E. coli* cells. **(C)** KEGG enrichment analysis of significant metabolic pathways resulting from SOD deficiency (the top 10 pathways are shown). G6P, glucose 6-phosphate; F6P, fructose 6-phosphate; FBP, fructose 1,6-bisphosphate; G3P, glyceraldehyde 3-phosphate; PEP, phosphoenolpyruvate; PYR, pyruvate; AceCoA, acetyl-CoA; CIT, citrate; ICIT, isocitrate; AKG, α -ketoglutaric acid; SuccCoA, succinyl-CoA; SUCC, succinate; FUM, fumarate; MAL, malate; OAA, oxaloacetate; 6PGL, 6-phospho-glucono-1,5-lactone; 6PG, gluconate 6-phosphate; RU5P, ribulose 5-phosphate; X5P, xylulose 5-phosphate; R5P, ribose-5-phosphate; PRPP, 5-phospho-α -D-ribose 1-diphosphate. Red font indicates significant up-regulation of protein expression; green font indicates significant down-regulation of protein expression. *P*-values and fold change for the proteins between the two groups were calculated using the R package *t*-test. A *p*-value < 0.05 is considered significant. PrsA* indicates that PrsA expression was elevated, although it was not statistically significant. The *p*-values and detailed fold changes are presented in Supplementary Table 1.

Superoxide is generated alongside oxidative phosphorylation (cellular respiration) (Imlay and Fridovich, 1991; Imlay, 2008). Under physiological conditions, SOD maintains superoxide at a level tolerable for bacterial cells (Gort and Imlay, 1998). Superoxide cannot pass through the cell membrane (Korshunov and Imlay, 2002). If highly active oxidative phosphorylation is maintained despite SOD defects, excessive intracellular superoxide accumulation may be lethal to bacterial cells (Fujii et al., 2022). We hypothesized that the SOD-deficient mutant could reduce superoxide production by decreasing oxidative phosphorylation. As expected, when *sodA*-*sodB* was deleted, the expression of oxidative phosphorylation Complex I (NADH dehydrogenase) proteins NuoABC, NuoE-I, and NuoLM, Complex II (succinate dehydrogenase) proteins SdhABC decreased (Figure 1A). Reduced expression of the ATP synthase subunits AtpA and AtpD indicates limited ATP synthesis (Figure 1A). These data indicate that *E. coli* can reduce superoxide production at the source when SOD is defective.

### 2.3 SOD deficiency promoted PPP metabolism and the glyoxylate cycle

Reduced levels of NADH dehydrogenase in the SOD-deficient mutant may reflect low levels of the reaction substrate NADH, which is typically derived from central carbon metabolism (Titov et al., 2016; Yang et al., 2020). We then analyzed the effect of SOD deficiency on central carbon metabolism. This work added glucose and amino acids to the basal salt medium as carbon and nitrogen sources (Morigen et al., 2005; Qiao et al., 2025b). Deletion of the *sodA*-*sodB* genes leads to reduced Pgi levels and increased Zwf expression, indicating that glucose-6-phosphate (G6P) is shifted toward the PPP metabolism (Figure 1B). Obviously, SOD deficiency leads to an overall upregulation of PPP metabolism-related protein levels (Figure 1B). TktAB and TalAB can feed PPP metabolites into glycolysis (Figure 1B). Expression of fructose-bisphosphate aldolase FabB is upregulated in glycolysis, whereas expression of the pyruvate dehydrogenase E1 and E2 subunits (AceE and AceF) is reduced in the SOD-deficient mutant (Figure 1B), inhibiting the conversion of pyruvate to acetyl-CoA. Acetyl-CoA is the initiating substrate of the TCA cycle, and we found that the expression of GltA, which mediates the generation of citrate from acetyl-CoA and oxaloacetate, was unexpectedly increased following the reduction of glycolytic sources of acetyl-CoA (Figure 1B). Interestingly, the glyoxylate cycle, typically characterized by high expression of AceA and AceB, was exacerbated by *sodA*-*sodB* deletion (Figure 1B). Meanwhile, lower expression of SdhABC and FumAB indicates reduced flux in the TCA cycle (Figure 1B).

### 2.4 SOD deficiency stimulated the fatty acid degradation pathway

The interference of SOD deficiency with central metabolism raises the question: acetyl-CoA is a substrate of the glyoxylate cycle; Why does the glyoxylate cycle surge when acetyl-CoA production from glycolysis is reduced (Figure 1B)? To answer this question, we performed KEGG pathway enrichment analysis of the proteomic data. Although fatty acids were not included in the medium (Qiao et al., 2025b), fatty acid degradation was emphasized in the KEGG enrichment (Figure 1C), and the levels of proteins like FadE, which are involved in fatty acid degradation, were overall upregulated after SOD defects (Figure 1D), leading to the production of acetyl-CoA (Pavoncello et al., 2022). This explains the phenomenon of a weaker glycolysis paired with a stronger glyoxylate cycle (Figure 1B).

### 2.5 SOD deficiency enhanced the synthesis of histidine, aromatic amino acids, and enterobactin

Additionally, the KEGG data were enriched for oxidative phosphorylation and glyoxylate metabolism (Figure 1C), consistent with our findings above (Figures 1A, B). Obviously, amino acid synthesis is present in the first and third positions of the enriched pathway (Figure 1C). In-depth analysis of protein expression profiles revealed that SOD deficiency stimulated histidine synthesis, as the expression of His proteins involved in histidine synthesis was upregulated (Figure 1E). 5-phospho-α-D-ribose 1-diphosphate (PRPP) serves as a substrate for histidine synthesis. We observe that in the SOD-deficient mutant, PrsA expression was increased in the PPP metabolism (Figure 1B), indicating that the conversion to PRPP is enhanced, thereby linking the PPP metabolism to histidine synthesis.

Moreover, the expression of Trp proteins and PheA, which are involved in tryptophan and phenylalanine synthesis, also increased (Figure 1F), suggesting that SOD deficiency might have elevated tryptophan and phenylalanine levels. The substrate chorismate, necessary for tryptophan and phenylalanine synthesis, can also originate from the PPP metabolism. High expression of Tkt, Tal, and Aro proteins can enhance chorismate production via this pathway (Figure 1F).

In addition to its role in amino acid synthesis, chorismate is also a substrate for enterobactin synthesis. SOD deficiency resulted in a significant increase in the biosynthesis of siderophore group non-ribosomal peptides (Figure 1B). In this pathway, all Ent proteins involved in enterobactin synthesis were upregulated (Figure 1G). These data indicate that SOD deficiency facilitates the PPP metabolism, stimulating enterobactin synthesis.

### 2.6 SOD deficiency makes *E. coli* more sensitive to hydrogen peroxide-mediated killing

**FIGURE 2:**
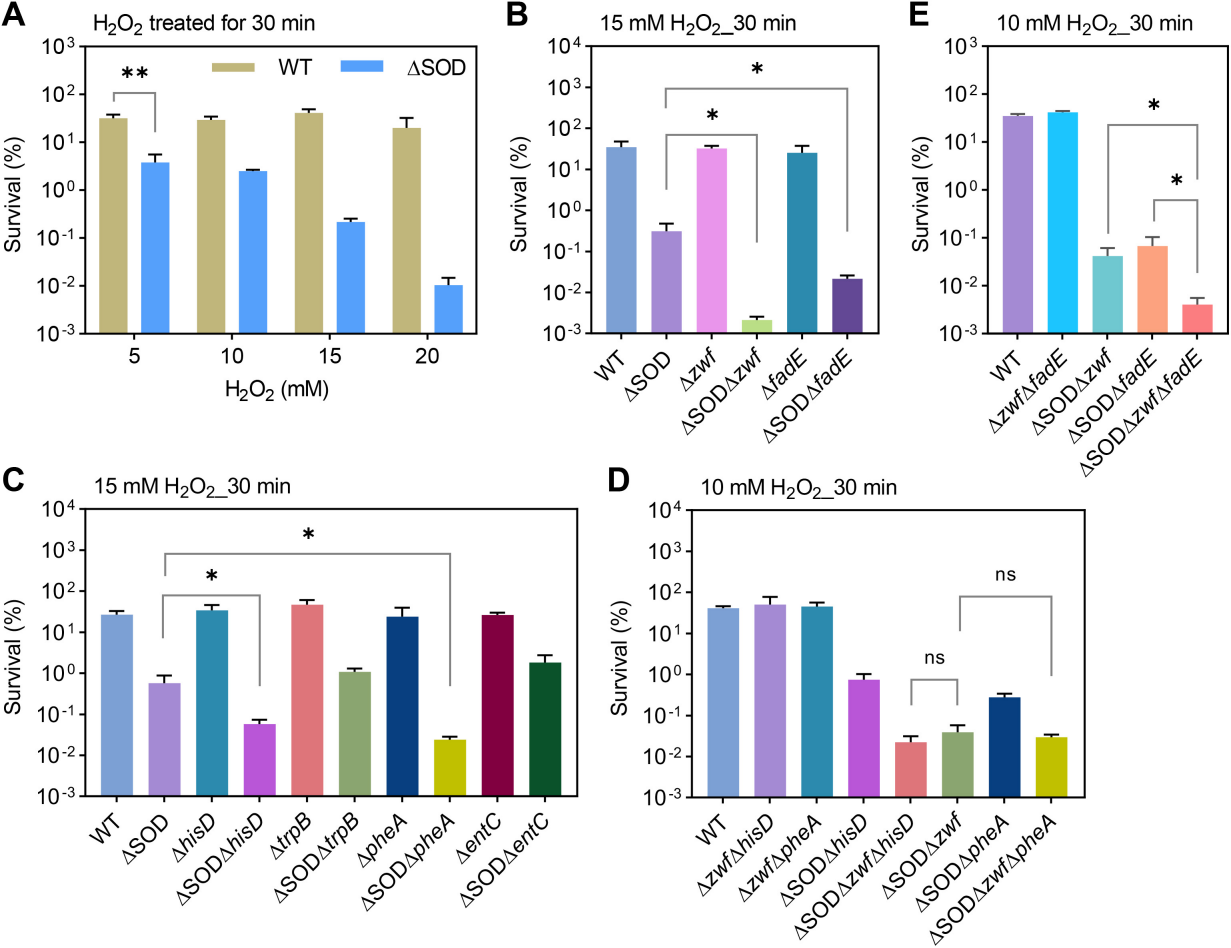
Survival of exponentially growing *E. coli* after hydrogen peroxide treatment. **(A)** Survival of WT and SOD mutant strains after 30 min of H_2_O_2_ treatment at the indicated concentration. **(B,C)** Survival of *E. coli* strains after 30 min of treatment with 15 mM H_2_O_2_. The hydrogen peroxide concentration was selected based on the results shown in panel **(A)**. **(D,E)** Survival of *E. coli* strains after 30 min of treatment with 10 mM H_2_O_2_. Given the potential additive sensitivity of multiple mutants to H_2_O_2_ stress, panels **(D,E)** used lower H_2_O_2_ concentrations than panels **(B,C)** to ensure that the CFU of *E. coli* remained within the detectable range after H_2_O_2_ treatment. Experiments were performed at least three times independently. Each plotted data point represents the mean ± SD. Data significance analysis was performed using a *t*-test (two-tailed, unpaired). *: 0.01 < *p*-value < 0.05; **: 0.001 < *p*-value < 0.01; ns: no significance.

The proteomics analysis above indicates that SOD deficiency induces oxidative stress regulation and metabolic reprogramming. Among these changes, stimulated PPP metabolism produces NADPH, a crucial intracellular antioxidant. How does this stress response influence cellular adaptation to additional oxidative stress? We found that exogenous hydrogen peroxide treatment at 5–20 mM for 30 min did not significantly reduce WT cell survival, but it decreased SOD-deficient mutant survival in a concentration-dependent manner (Figure 2A). This indicates that insufficient regulation of oxidative stress, induced prematurely by SOD deficiency, fails to help bacteria cope with additional oxidative stress.

### 2.7 PPP metabolism and fatty acid degradation pathway contribute to antioxidant defense in the SOD-deficient mutant

Next, we genetically validated whether SOD-deficient metabolic reprogramming contributes to antioxidant defense. A single deletion of *zwf*, which blocks the PPP pathway, did not alter *E. coli* survival after 30 min of 15 mM H_2_O_2_ treatment but increased the sensitivity of SOD mutants to H_2_O_2_ killing (Figure 2B), indicating the antioxidant role of PPP metabolism in SOD deficiency.

When SOD is deficient, the synthesis of histidine, aromatic amino acids, and enterocystine increases (Figures 1E–G). To determine whether these pathways contribute to antioxidant defense or merely serve as passively stimulated downstream components of the PPP (Figures 1E–G), we also performed knockouts of the corresponding synthetic genes. We found that deleting *hisD* or *pheA*–which disrupts histidine and phenylalanine synthesis, respectively–increased the hydrogen peroxide sensitivity of the SOD-deficient mutant, whereas deleting *trpB* or *entC* had no effect on hydrogen peroxide sensitivity in either the WT or SOD mutant strain (Figure 2C). Furthermore, deleting *hisD* or *pheA* in the absence of *zwf* did not increase the hydrogen peroxide sensitivity of the ΔSODΔ*zwf* mutant (Figure 2D), suggesting that PPP-derived substrates are important for the antioxidant capacity conferred by the histidine and phenylalanine synthesis pathways.

Superoxide dismutase deficiency stimulated fatty acid degradation pathway (Figure 1D). Deletion of *fadE* blocks fatty acid degradation without altering WT *E. coli* survival after 30 min of 15 mM H_2_O_2_ treatment, but it increases the sensitivity of the ΔSOD mutant to H_2_O_2_ killing (Figure 2B). Unlike the clear upstream-downstream relationship between aromatic amino acid and enterobacterial synthesis and PPP metabolism, fatty acid degradation operates relatively independently of PPP metabolism. Furthermore, the combined absence of *zwf* and *fadE* amplifies the sensitivity of SOD mutants to H_2_O_2_ killing (Figure 2E), indicating that PPP metabolism and fatty acid degradation pathway jointly contribute to the antioxidant capacity of SOD-deficient mutants.

## 3 Discussion

Under physiological conditions, active cellular respiration provides energy for rapid multiplication and is linked to the production of the toxic metabolite superoxide. SOD is considered the first line of defense against endogenous oxidative stress in bacteria and can convert superoxide to hydrogen peroxide. In addition to the role of catalase in decomposing H_2_O_2_ into H_2_O, H_2_O_2_ also participates in a Fenton reaction with iron ions, generating highly oxidizing hydroxyl radicals (Imlay, 2003, 2008). Alongside antioxidant systems, several damage repair mechanisms exist to ensure the survival and genetic stability of rapidly growing bacteria. When SOD is defective, intracellular superoxide levels rise, inducing oxidative stress (Imlay and Fridovich, 1991). Thus, the SOD-deficient mutant serves as a model for studying bacterial oxidative stress defense networks and survival strategies.

Beyond superoxide, SOD deficiency led to various forms of reactive oxygen stress. This is indicated by the increased expression of the catalase KatE and the organic peroxidases Tpx and BtuE (Supplementary Figure 1). SOD defects promoted fatty acid degradation pathway (Figure 1D), a process reported to produce H_2_O_2_ (Sirithanakorn and Imlay, 2024), which may explain the elevated katE levels. The elevated expression of the lipid peroxidase Tpx (Supplementary Figure 1), along with PlsC and ClsB (Supplementary Table 2), which are involved in lipid synthesis (Coleman, 1990; Jeucken et al., 2018), suggests that SOD deficiency induces oxidative damage to lipids. Lipid peroxidation results in a variety of toxic peroxides, including malondialdehyde (MDA) and 4-hydroxynon-enal (4-HNE) (Ayala et al., 2014). The action of superoxide on iron-sulfur clusters results in the release of free iron, and SOD is also considered a metal ion chelator (Culotta, 2000); therefore, SOD defects may elevate free iron levels. When H_2_O_2_ and iron ions are present, a Fenton reaction takes place that generates hydroxyl radicals. Additionally, membrane lipid peroxidation generates singlet oxygen, which possesses strong oxidizing properties (Boveris et al., 1980). Overall, SOD deficiency results in elevated superoxide levels and indirectly contributes to a complex state of oxidative stress in bacteria.

Superoxide dismutase deficiency induces antioxidant metabolic reprogramming. The PPP pathway is globally upregulated in SOD-deficient cells, and its metabolites serve as substrates for histidine and phenylalanine synthesis, thereby activating amino acid synthesis pathways (Figures 1B, E, F). The antioxidant capacity of PPP metabolism primarily stems from the production of the antioxidant NADPH (Sandoval et al., 2011). When the PPP function is fully preserved, but downstream amino acid synthesis is impaired, the antioxidant capacity of SOD mutants decreases (Figure 2C). These data reveal a combined antioxidant pathway involving PPP and downstream histidine and phenylalanine synthesis.

Previous studies have reported that supplementing with aromatic amino acids promotes the growth of SOD mutant (Carlioz and Touati, 1986). In this study, casein hydrolysate was added to the medium, thus excessive histidine and phenylalanine may be present during the bacterial exponential growth phase. We hypothesize that the antioxidant effect arises from the amino acid synthesis process or other intermediate metabolic pathways, rather than from the final products, histidine or phenylalanine. As anticipated, supplemental histidine and phenylalanine did not help wild-type or SOD mutant strains counteract hydrogen peroxide toxicity (Supplementary Figure 2). How the histidine and phenylalanine synthesis pathways specifically confer antioxidant effects remains to be demonstrated with further biochemical evidence.

In addition to stimulating PPP to enhance antioxidant capacity, SOD deficiency inhibits the conversion of pyruvate derived from glycolysis to acetyl-CoA, thereby weakening TCA cycle flux. This reduces NADH production, a key respiratory substrate, thereby diminishing oxidative phosphorylation and superoxide generation (Figure 1B).

During central carbon metabolism reprogramming in response to oxidative stress, SOD deficiency exacerbates the glyoxylate cycle and fatty acid degradation pathway. Indeed, combined fatty acid degradation and glycolysis can provide carbon sources for bacterial growth during carbohydrate starvation (Rabin et al., 1965). When SOD was deficient, glucose metabolism shifted more toward the PPP pathway, potentially creating an illusion of carbon-source scarcity and triggering fatty acid degradation pathway (Figures 1B, D). In this study, the medium lacked fatty acids. Consistent with this, the SOD defect did not induce overexpression of FadD, a protein responsible for converting oleate to oleoyl-CoA. Therefore, activation of the fatty acid degradation pathway in SOD deficiency may serve as a feedback mechanism to reduce acetyl-CoA levels from glycolysis. Overall, our work provides a proteomics perspective on metabolic reprogramming induced by SOD deficiency; subsequent metabolomics analysis may offer further details. Given the potential impact of differences in the nutrient composition of culture media on bacterial metabolism, future studies comparing metabolic reprogramming pathways induced by SOD deficiency under varying nutritional conditions would be meaningful for further identifying the core antioxidant metabolic pathways in SOD-deficient cells.

In summary, our work reveals that SOD deficiency induces antioxidant strategies in advance, but is more sensitive to hydrogen peroxide. These antioxidant regulations enable *E. coli* cells to survive under SOD deficiency but do not provide additional antioxidant support, suggesting a balanced strategy for stress resistance and growth. Among them, metabolic reprogramming involving central carbon metabolism, oxidative phosphorylation, amino acid metabolism, and fatty acid degradation pathways constitutes a crucial strategy for SOD-deficient cells to cope with additional oxidative stress.

## 4 Materials and methods

### 4.1 Bacterial strains, reagents, and medium

*Escherichia coli* K-12 strains used in the study are listed in Supplementary Table 3. The gene knockout mutants were constructed using CRISPR following the method described by Jiang et al. (2015). Briefly, the sgRNA fragment and homology arm fragments were amplified using the primers listed in Supplementary Table 4 and inserted into the pTargetF plasmid. The recombinant pTargetF plasmid was then transformed into the parental strain harboring the pCas9 plasmid. Positive clones were selected on kanamycin and spectinomycin plates. After confirming the correct gene knockout by PCR and sequencing, the pTargetF and pCas9 plasmids were eliminated by supplementing with IPTG and by high-temperature treatment, respectively. Histidine hydrochloride monohydrate and L-phenylalanine were purchased from Sangon Biotech Inc., (Shanghai, China). The ABTG-Casamino acid (ABTG-CAA) medium consists 6 g/L Na_2_HPO_4_, 2 g/L (NH_4_)_2_SO_4_, 3 g/L KH_2_PO_4_, 3 g/L NaCl, 10 mg/L vitamin B1, 3 μM FeCl_3_, 0.1 mM CaCl_2_, 1 mM MgCl_2_, 0.2% glucose, and 0.5% Casamino acid.

### 4.2 Proteomics analysis

*Escherichia coli* strains BW25113 and Δ*sodA*Δ*sodB* were incubated overnight in LB medium, then diluted 1:5000 into fresh ABTG-CAA medium and cultured to OD_450_ = 0.15–0.2 (exponential growth phase). Afterward, the samples were collected by centrifugation, flash-frozen in liquid nitrogen, and stored at −80 °C before being shipped to Majorbio (Shanghai, China) for total protein extraction, library construction, and proteomic analysis. More details are available in our previous report (Qiao et al., 2025b).

The proteomics data have been deposited at the Integrated Proteome Resources^1^ with the ID IPX0009699000 (ProteomeXchange ID: PXD055835) (Qiao et al., 2025b). In the UniProt database, a protein may have multiple peptide entries (each with a different accession number); therefore, the protein expression profiles obtained may indicate that the same protein name corresponds to multiple expression values. When analyzing the data, if this situation arises, the expression of the longer peptide with higher abundance is used to represent the corresponding protein. Peptide data detected in only one sample (Δ*sodA*Δ*sodB* or WT) were excluded to ensure data reliability.

### 4.3 Hydrogen peroxide-mediated killing

*Escherichia coli* cells were cultured overnight at 37 °C and 200 rpm in LB medium, then diluted 1:5000 into ABTG-CAA medium and cultured to an OD_450_ of 0.2 (exponential growth phase). Subsequently, hydrogen peroxide was added for treatment. After 30 min of hydrogen peroxide treatment, take equal-volume samples and perform a 10-fold serial dilution using 0.9% saline solution. Then dispense 10 μL of the diluted sample onto LB agar plates, performing three technical replicates per sample. After incubating at 37 °C for 16 h, count the colony-forming units (CFU) and calculate the survival relative to untreated culture samples.

## Data Availability

The original contributions presented in this study are included in this article/Supplementary material, further inquiries can be directed to the corresponding authors.

## Footnotes

1
https://www.iprox.org
