# Supplementary material for: Despite inducing antioxidant regulation, superoxide dismutase deficiency makes Escherichia coli more sensitive to hydrogen peroxide
Source: Front Microbiol. 2026 Mar 10;17:1793871. doi: 10.3389/fmicb.2026.1793871 (PMC13010160; doi:10.3389/fmicb.2026.1793871)
Supplement: Supplementary file 1 [file Data_Sheet_1.pdf]

Supplementary Information

Despite inducing antioxidant regulation, superoxide dismutase deficiency makes *Escherichia coli* more sensitive to hydrogen peroxide

Yuejuan Nong[1†], Jiaxin Qiao[2†], Yixuan Zhao[1], Jingjing Wang[3], Li Xin[4], Weijie Wang[3]* and Weiwei Zhu[1]*

[1] State Key Laboratory of Vaccines for Infectious Diseases, Xiang-An Biomedicine Laboratory, National Innovation Platform for Industry-Education Integration in Vaccine Research, Department of Laboratory Medicine, School of Public Health, Xiamen University, Xiamen, China.

[2] Inner Mongolia Key Laboratory for Molecular Regulation of the Cell, School of Life Sciences, Inner Mongolia University, Hohhot, China.

[3] Research Center for Clinical Medicine, The First Affiliated Hospital of Kunming Medical University, Kunming, Yunnan, China.

[4] Department of Clinical Pharmacy, The Affiliated Hospital of Yunnan University, Kunming, China.

[†] These authors have contributed equally to this work.

*Address correspondence to Weiwei Zhu, wwzhu91@xmu.edu.cn; Weijie Wang, wangweijie@kmmu.edu.cn.

Table of Contents

1. Supplementary Figures
2. Supplementary Tables
3. Supplementary references

Supplementary Figures

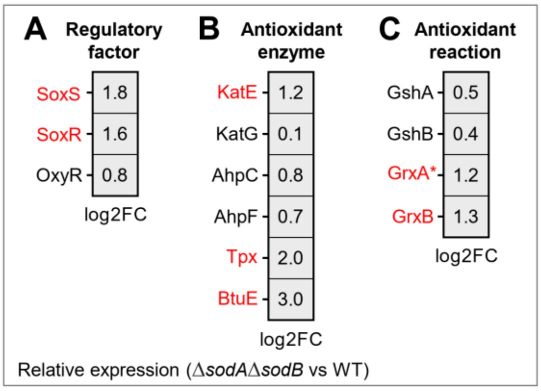

Supplementary Figure 1. The effect of SOD deficiency on the expression of oxidative stress regulators, antioxidant enzymes, and proteins related to antioxidant reactions. Red font indicates significant up-regulation of protein expression; black font represents changes in protein expression without significant differences; FC, fold change. *p*-values and fold change for the proteins between the two groups were calculated using the R package *t*-test. A *p*-value < 0.05 is considered significant. The *p*-values and detailed fold changes are presented in Supplementary Table S1. In panel C, GrxA* indicates that GrxA expression was elevated, although it was not statistically significant.

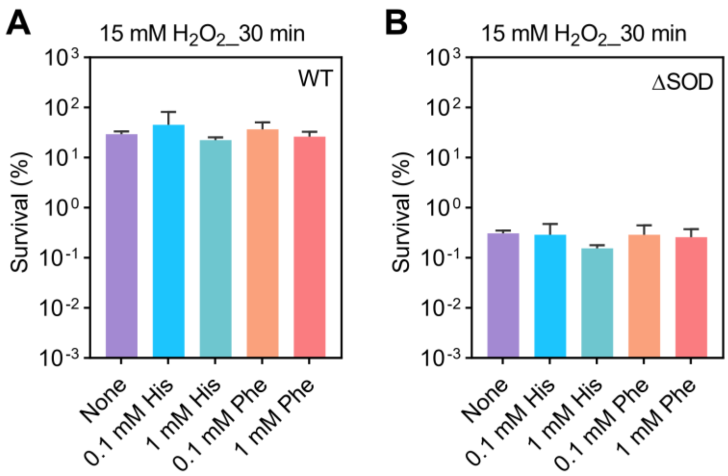

Supplementary Figure 2. Survival of exponentially growing *E. coli* after hydrogen peroxide treatment. Survival of WT (**A**) and SOD mutant (**B**) strains after 30 minutes of H_2O_2 treatment at the indicated concentration. Exponentially growing cells were pretreated with the indicated concentrations of histidine or phenylalanine for 30 min, followed by a 30-min exposure to hydrogen peroxide. His, Histidine hydrochloride monohydrate**;** Phe, Phenylalanine**.** Experiments were performed at least three times independently. Each plotted data point represents the mean ± SD.

Supplementary Tables

Supplementary Table 1. Differential protein expression caused by the *sodA*-*sodB* deletion is related to Figure 1.

Uniprot accession ID	Protein name	Log2FC (*sodAB*/WT)[1]	*p*-values
A0A1M1HXA4	SoxS	1.802	0.007679
A0A085PAQ1	SoxR	1.573	0.04801
A0A0D8WE52	OxyR	0.7638	0.01514
A0A037YQJ9	KatE	1.196	0.006189
A0A0E1M4A2	KatG	0.1057	0.1545
A0A5B9AKJ5	AhpC	0.763	0.06513
A0A0D8W8T6	AhpF	0.7321	0.01923
A0A5B9AWF1	Tpx	1.971	0.004006
A0A377BCK4	BtuE	3.02	0.01312
A0A0B0VPN8	GshA	0.519	0.1273
A0A0A0FH38	GshB	0.3763	0.06028
A0A6D0L2T1	GrxA	1.217	0.1048
A0A080J6I4	GrxB	1.333	0.000552
A0A080IXW7	NuoA	-1.331	0.002184
A0A376D7L1	NuoB	-2.281	0.003063
P33599	NuoC	-1.48	0.002015
A0A3L5PAE6	NuoE	-2.104	0.000546
A0A0E2L5S1	NuoF	-1.97	0.002235
A0A828U7K8	NuoG	-2.344	0.000369
A0A0E2L5F5	NuoH	-1.499	0.0479
A0A376RG70	NuoI	-2.119	0.00027
A0A080IR16	NuoL	-1.161	0.03423
A0A066T8Y6	NuoM	-3.231	0.004925
A0A0D8WG58	SdhA	-1.198	0.004468
A0A8G8RZ87	SdhB	-1.674	0.01411
A0A0F3VL59	SdhC	-1.41	0.01535
A0A3W5Y2S6	AtpA	-3.443	0.01953
A0A3W5Y2S6	AtpD	-3.443	0.01953
A0A6D0EIV3	Pgi	-3.755	0.009158
A0A2X1MYA3	FbaB	3.201	0.000966
A0A376VXZ2	AceE	-1.097	0.002911
A0A377N397	AceF	-2.5	0.04672
A0A2X1KQC7	Zwf	1.261	0.000704
A0A0L7AHF5	Pgl	1.322	0.02231
A0A0K4UVT9	Gnd	1.081	0.008801
D8E2M2	TktA/B	1.863	0.0039
A0A7A2WVI5	TalA/B	2.021	0.006194
A0A376KZ28	PrsA	1.321	0.1547
A0A080JC36	GltA	1.286	0.001831
A0A1U9T3C0	FumA	-1.59	0.000686
P14407	FumB	-1.625	0.001463
A0A0H0I093	FumC	2.155	0.00396
A0A0A0H7X0	Mqo	-1.108	0.003388
A0A891SHB6	Mdh	1.055	0.004693

Continued Supplementary Table 1.			
A0A5N8HJB5	AceA	1.13	0.004952
A0A376U0Y9	AceB	1.567	0.03943
A0A376U3I3	AcnB	-1.278	0.01592
A0A377E1M8	FadE	3.76	0.02507
A0A3L2NQZ4	FadJ	1.572	0.000536
A0A093FWZ7	FadB	1.349	0.002544
A0A0E1T582	FadA	1.952	0.001025
A0A0B0SN95	FadI	1.379	0.02188
A0A2X1KS60	HisG	1.067	0.01303
A0A061KIM8	HisI	1.308	0.0477
A0A0A0FEG8	HisH	1.331	0.03192
A0A827HQN1	HisB	1.642	0.001314
A0A024L151	HisC	1.485	0.0393
A0A376U6T9	HisD	2.297	0.000669
A0A376LAI3	AroF	1.443	0.02917
A0A066T4M3	AroA	2.804	0.02573
A0A0C2AMW1	AroD	1.86	0.007991
J7QQC1	TrpD	2.122	0.002524
A0A7Z8HVD2	TrpE	2.529	0.002217
A0A377D334	TrpC	1.931	0.004618
A0A0J2BLC5	TrpA	2.399	0.000127
A0A376KV38	TrpB	1.863	0.01731
A0A066Q2A1	PheA	1.304	0.00557
A0A066SXR9	EntC	2.162	0.03019
A0A377DX70	EntB	2.304	0.007703
A0A2X1Q0C8	EntA	3.669	0.03007
A0A0E1T0G8	EntD	3.745	0.000509
A0A0B0VU53	EntE	2.235	0.001924
A0A0K4SZL7	EntF	1.674	0.002995
[1] FC: fold change; *sodAB*: Δ*sodA*Δ*sodB*.			

Supplementary Table 2. Differential protein expression caused by the *sodA*-*sodB* deletion.

**Protein**	**Protein function**	**Log2FC (*sodAB*/WT)**[1]	***p*-values**
PlsC	1-acylglycerol-3-phosphate *O*-acyltransferase	1.2	0.031
ClsB	cardiolipin synthase	1.6	0.015

[1] FC: fold change; *sodAB*: Δ*sodA*Δ*sodB*.

Supplementary Table 3. Bacterial strains used in the study.

Strain	genotype	Source/reference
BW25113	*E. coli* wild-type *rrnB*3 Δ*lacZ*4787 *hsdR*514 Δ(*araBAD*)567 Δ(*rhaBAD*)568 *rph-1*	(Baba et al., 2006)
Δ*sodA*Δ*sodB*	ΔSOD, deletion of the *sodA* and *sodB* genes in the BW25113 chromosome	(Qiao et al., 2025)
Δ*zwf*	Deletion of the *zwf* gene in the BW25113 chromosome	This work
ΔSODΔ*zwf*	Deletion of the *zwf* gene in the ΔSOD chromosome	This work
Δ*fadE*	Deletion of the *fadE* gene in the BW25113 chromosome	This work
ΔSODΔ*fadE*	Deletion of the *fadE* gene in the ΔSOD chromosome	This work
ΔzwfΔ*fadE*	Deletion of the *fadE* gene in the Δ*zwf* chromosome	This work
ΔSODΔ*zwf*Δ*fadE*	Deletion of the *fadE* gene in the ΔSODΔ*zwf* chromosome	This work
Δ*hisD*	Deletion of the *hisD* gene in the BW25113 chromosome	This work
ΔSODΔ*hisD*	Deletion of the *hisD* gene in the ΔSOD chromosome	This work
Δ*trpB*	Deletion of the *trpB* gene in the BW25113 chromosome	This work
ΔSODΔ*trpB*	Deletion of the *trpB* gene in the ΔSOD chromosome	This work
Δ*pheA*	Deletion of the *pheA* gene in the BW25113 chromosome	This work
ΔSODΔ*pheA*	Deletion of the *pheA* gene in the ΔSOD chromosome	This work
Δ*entC*	Deletion of the *entC* gene in the BW25113 chromosome	This work
ΔSODΔ*entC*	Deletion of the *entC* gene in the ΔSOD chromosome	This work
Δ*zwf*Δ*hisD*	Deletion of the *hisD* gene in the Δ*zwf* chromosome	This work
Δ*zwf*Δ*pheA*	Deletion of the *pheA* gene in the Δ*zwf* chromosome	This work
ΔSODΔ*zwf*Δ*hisD*	Deletion of the *hisD* gene in the ΔSODΔ*zwf* chromosome	This work
ΔSODΔ*zwf*Δ*pheA*	Deletion of the *pehA* gene in the ΔSODΔ*zwf* chromosome	This work

Supplementary Table 4. Primers used in the study.

Primer Name	Sequence（5′--3′）	Usage
entC-sgRNA-F	GGACTAGTACGGTTGATTGCGCAAAACC GTTTTAGAGCTAGAAATAGC	To amplify *entC*-specific sgRNA
sgRNA-R	CTCAAAAAAAGCACCGACTCGG	To amplify specific sgRNA
entC-HA-up-F	CCGAGTCGGTGCTTTTTTTGAG GACAAAGCGCACAATCCGTC	To amplify *entC* upstream homology arm fragment
entC-HA-up-R	ACGTTCAACATGGTAGAAAG GGTCTGCTGTACTTCCTCAG	
entC-HA-dn-F	CTGAGGAAGTACAGCAGACC CTTTCTACCATGTTGAACGT	To amplify *entC* downstream homology arm fragment
entC-HA-dn-R	AACTGCAGTTTCAGCAGCGCGAAAAAGG	
entC-check-up	CTGAACTGCGGCTATTCCTG	Upstream primer for *entC* identification
entC-check-dn	TTTCAGCAGCGCGAAAAAGG	Downstream primer for *entC* identification
fadE-sgRNA-F	GGACTAGTACCCTGCCTTCCAACTCAAC GTTTTAGAGCTAGAAATAGC	To amplify *fadE*-specific sgRNA
fadE-HA-up-F	CCGAGTCGGTGCTTTTTTTGAG GCTGAATGGCGTGAATATTG	To amplify *fadE* upstream homology arm fragment
fadE-HA-up-R	TTCCGCACTTTCTCCGGCAA CAGGACAACCGTAGCGAGAA	
fadE-HA-dn-F	TTCTCGCTACGGTTGTCCTG TTGCCGGAGAAAGTGCGGAA	To amplify *fadE* downstream homology arm fragment
fadE-HA-dn-R	AACTGCAGGCACAAACCGTAACGTTGGG	
fadE-check-up	GACGGTTTTCACGGTAGCGA	Upstream primer for *fadE* identification
fadE-check-dn	TGGCGCAGGATGATTTAACC	Downstream primer for *fadE* identification
hisD-sgRNA-F	GGACTAGTTGCCGATGAGATCCTTATG GTTTTAGAGCTAGAAATAGC	To amplify *hisD*-specific sgRNA
hisD-HA-up-F	CCGAGTCGGTGCTTTTTTTGAG GATGGCGAAATGGAAGAATC	To amplify *hisD* upstream homology arm fragment
hisD-HA-up-R	AGGGCGTTAACACGCAAAGT ACAGCTATTCCAGTCAATGA	
hisD-HA-dn-F	TCATTGACTGGAATAGCTGT ACTTTGCGTGTTAACGCCCT	To amplify *hisD* downstream homology arm fragment
hisD-HA-dn-R	CCCAAGCTTGTAGAGGATGGCGTCTTTAC	
hisD-check-up	GAACGGTTCTGTTGAAGTCG	Upstream primer for *hisD* identification
hisD-check-dn	TGTCGGAAATGCCCTGTAAG	Downstream primer for *hisD* identification
pheA-sgRNA-F	GGACTAGTCATACCAGCTTGTCGATTGT GTTTTAGAGCTAGAAATAGC	To amplify *pheA*-specific sgRNA
pheA-HA-up-F	CCGAGTCGGTGCTTTTTTTGAG CCGCAACATCGGTGAAAGAC	To amplify *pheA* upstream homology arm fragment
pheA-HA-up-R	GGCACTACGTTCTCACTTGGGATTTTCTCTC GCAGCGCCA	
pheA-HA-dn-F	TGGCGCTGCGAGAGAAAATC CCAAGTGAGAACGTAGTGCC	To amplify *pheA* downstream homology arm fragment

Continued Supplementary Table 4.		
pheA-HA-dn-R	AACTGCAG TCAGGATCCGCAGCTTTATG	
pheA-check-up	CAATACACCTAACGGCGTTC	Upstream primer for *pheA* identification
pheA-check-dn	TGCTACTTTTGCTTACGGGC	Downstream primer for *pheA* identification
trpB-sgRNA-F	GGACTAGTCGAAACCGCGCACTATATGC GTTTTAGAGCTAGAAATAGC	To amplify *trpB*-specific sgRNA
trpB-HA-up-F	CCGAGTCGGTGCTTTTTTTGAG CTGGAAAGCATTAAGCGTCG	To amplify *trpB* upstream homology arm fragment
trpB-HA-up-R	CGTGCTTTCAAAATATCGTG GCCAAACTCACCAAAATAGG	
trpB-HA-dn-F	CCTATTTTGGTGAGTTTGGC CACGATATTTTGAAAGCACG	To amplify *trpB* downstream homology arm fragment
trpB-HA-dn-R	AACTGCAG CAATGCCTTTGTTAAACACC	
trpB-check-up	ATGTGGTGGACAAAGCTAAG	Upstream primer for *trpB* identification
trpB-check-dn	GTGCGACATTATGACGCAAC	Downstream primer for *trpB* identification
zwf-sgRNA-F	GGACTAGTAATTTGCAAAGGGCTTGGCGGT TTTAGAGCTAGAAATAGC	To amplify *zwf*-specific sgRNA
zwf-HA-up-F	CCGAGTCGGTGCTTTTTTTGAGGAA TGGATCGCGTTATCGGG	To amplify *zwf* upstream homology arm fragment
zwf-HA-up-R	CATAAAGGATAAGCGCCT TAAGTTAACTAACCCG	
zwf-HA-dn-F	CGGGTTAGTTAACTTAAG GCGCTTATCCTTTATG	To amplify *zwf* downstream homology arm fragment
zwf-HA-dn-R	AACTGCAGCGATATTGTTACGCAAC	
zwf-check-F	GTTCGCTAACATTGGCTTCC	Upstream primer for *zwf* identification
zwf-check-R	GAGCATGTCGTTATAGGAGG	Downstream primer for *zwf* identification

Supplementary references

Baba, T., Ara, T., Hasegawa, M., Takai, Y., Okumura, Y., Baba, M., Datsenko, K.A., Tomita, M., Wanner, B.L., and Mori, H. (2006). Construction of *Escherichia coli* K-12 in-frame, single-gene knockout mutants: the Keio collection. *Mol Syst Biol* 2**,** 2006.0008.

Qiao, J., Zhu, W., Du, D., and Morigen, M. (2025). Characterizing Common Factors Affecting Replication Initiation During H_2O_2 Exposure and Genetic Mutation-Induced Oxidative Stress in *Escherichia coli*. *Int J Mol Sci* 26.
